# Temporal properties of the speed-accuracy trade-off for arm-pointing movements in various directions around the body

**DOI:** 10.1371/journal.pone.0291715

**Published:** 2023-09-21

**Authors:** Soma Okuuchi, Keisuke Tani, Keisuke Kushiro

**Affiliations:** 1 Graduate School of Human and Environment Studies, Kyoto University, Kyoto, Japan; 2 Faculty of Psychology, Otemon Gakuin University, Osaka, Japan; Institute of Psychology Chinese Academy of Sciences, CHINA

## Abstract

Human body movements are based on the intrinsic trade-off between speed and accuracy. Fitts’s law (1954) shows that the time required for movement is represented by a simple logarithmic equation and is applicable to a variety of movements. However, few studies have determined the role of the direction in modulating the performance of upper limb movements and the effects of the interactions between direction and distance and between direction and target size. This study examined the variations in temporal properties of the speed-accuracy trade-off in arm-pointing movements that directly manipulate objects according to the direction, distance, and target size. Participants performed pointing movements to the targets with 3 different sizes presented at 15 locations (5 directions and 3 distances) on a horizontal plane. Movement time (MT) for each trial in each condition was obtained. Subsequently, Mackenzie’s model (1992), MT = a + b log_2_(D/W +1), where D and W represent the distance and width of the target, respectively, was fitted. The slope factor b, a fitted parameter in the equation, was calculated and evaluated according to the changes in the direction, distance, and target size. The results showed that MTs exhibited anisotropy in the hemifield, being the smallest in the right-forward direction. Additionally, the slope factor b, as a function of distance, was smaller in the rightward direction than in the forward and left-forward directions. These results suggest that the degree of difficulty of upper limb movements expands heterogeneously in various directions around the body.

## Introduction

The fundamental principle behind human body movements is based on the trade-off between speed and accuracy. Fitts [1] originally stated that the movement time (MT) for pointing tasks can be explained by a simple formula using distance and target width. Fitts’s original model concerned one-dimensional movements of the upper limb, where the hand traveled between targets placed on the left and right sides. Mackenzie referred to Sharon’s theorem [2,3] and proposed an extended model (Eq 1), which is applicable to movements in various directions.


$$MT=a+b\;log_{2}(D/W+1)$$ (1)

Where D and W denote the distance and target width, respectively, and a and b denote the intercept and slope factor, respectively, depending on the conditions. The equation further claims that MT is linearly related to the term of log_2_(D/W+1) which is the so-called “Index of difficulty (ID)” indicating degree of difficulty for executing movements. Importantly, the equation explains that MT is determined by two parameters, distance and target width. Mackenzie’s model modified ID in Fitts’s model by introducing the effect of the target width based on the direction [3]. Likewise, many studies have elucidated the features and applications of Fitts’s law after it was published [4].

Several studies have been conducted to elucidate the nature of the speed-accuracy trade-off for pointing movements in various directions on a display with interface devices such as a joystick [5], mouse [6], and haptic robots [7]. However, few studies have examined upper limb movements that directly manipulate objects. Such movements, directly manipulating objects, differ in several senses from those using interface devices. Firstly, upper limb movements that directly manipulate objects travel a much longer distance (by several tens of centimeters) compared to those using devices, which travel only a few centimeters [8]. This produces a discrepancy in kinematic parameters, such as maximum velocity and acceleration. Secondly, the trajectories of movements with input devices, such as a mouse, are more linear on a plane [9], whereas those with the upper limbs are more curvilinear [10] with complex spatial features in three-dimension. Finally, the cognitive aspect of the movements is quite different because the manipulation of the cursor on a display requires an imaginal transformation between the coordinates where the interface device moves and where the cursor moves. Therefore, we hypothesized that upper limb movements that directly manipulate objects are affected by direction more than movements using interface devices. Based on this hypothesis, we examined the effects of direction on the speed-accuracy trade-off during arm-pointing movements.

Some previous studies have elucidated the effects of movement direction on the performance of upper limb movements [11,12]. Murata and Iwase [11] examined MTs during arm-pointing movements in relation to the direction of movement in which participants pointed to one of eight targets on a vertical plane. They observed anisotropy in MTs according to the direction of pointing movements and suggested a modified model concerning it. However, their study did not treat the distance and target size independently to compose the IDs. Several previous studies have elucidated the effects of distance and target size on the performance of upper limb movements [13,14]. It is known that distance has a greater impact on MTs than target size. For example, when pointing movements are executed using a finger, MTs are more sensitive to changes in distance than to target size [13,14]. However, the effects of the interaction between direction and distance and the interaction between direction and target size on arm-pointing movements are unclear. Considering the differences in the effects of distance and target size on the MT, we hypothesized that the interaction between direction and distance affects the performance of arm-pointing movements more than the interaction between direction and target size.

In addition to the MT, reaction time (RT) that represents the time required to initiate the movement was obtained in each condition. RT is influenced by cognitive and motor factors such as the motor planning and motor cost. Since these factors are modulated by direction [15,16], we supplementally examined how the initiation of upper limb movements depended on the direction.

From these perspectives, this study examined temporal properties of arm-pointing movements in the hemifield around the body under the independent manipulation of direction, distance, and target size. Subsequently, MTs obtained were fitted by Mackenzie’s equation (Eq 1) for each direction and the slope factor b, indicating a ratio of change in MTs in response to changes in distance and target size, was obtained. We hypothesized that MTs and RTs changed depending on the direction, and that the interaction between direction and distance affected MTs and the slope factor b more strongly than that between direction and target size.

## Method

### Participants

Twelve right-handed healthy volunteers (nine males and three females, aged 21–25 years) participated in the experiments conducted from February 6 to February 22, 2023. Before the experiment, all participants gave written informed consent. This study was approved by the Ethics Committee of the Graduate School of Human and Environmental Studies at Kyoto University. All the procedures were conducted in accordance with the Declaration of Helsinki (2013).

### Experimental set-up

**Fig 1:**
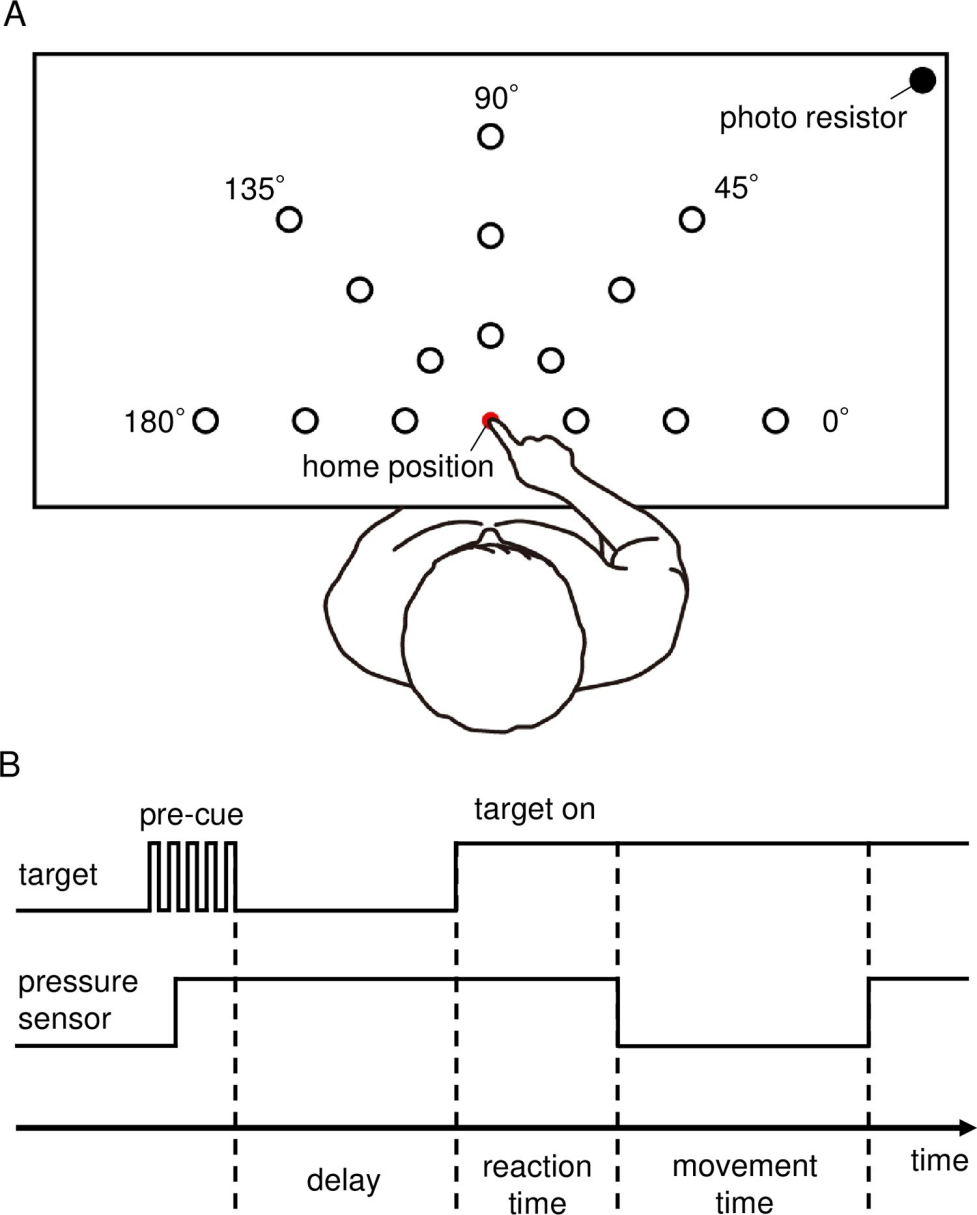
Schematic diagram of experimental setup and procedure. A: Experimental setup. Participants performed a pointing task on one of the targets presented in the workspace in front of their body. B: Experimental procedure. After pre-cueing and delay, participants reached the target with their right index finger as quickly as possible.

The participants sat on a chair in a dim room (Fig 1). A display (BRAVIA KDL-52HX900, SONY) 124 × 65 cm in width and depth was placed horizontally in front of the body for the target presentation. A photo-resistor (MI527, Macron International Groups) attached close to the boundary of the display area served to detect the onset of the target presentation by sensing a visual signal presented at the same time as the target presentation. A pressure sensor (FSR 400, Interlink Electronics) with a circular sensing component, 7 mm in diameter, was attached to the pad of the right index finger to detect contact with the display surface. A microcomputer (Arduino UNO) received an electric signal from the photoresistor or pressure sensor, and an infrared light-emitting diode (iLED) for each signal was switched on. A reflective marker with a diameter of 3 mm was attached to the right index finger of each participant. Three-dimensional position of the marker was recorded using a motion-capture system consisting of six cameras (OptiTrack Flex3, Natural Point) at a sampling rate of 100 Hz. In addition, the iLEDs indicating the target presentation and finger contact on the display were captured in the system.

### Task conditions and procedures

**Fig 2:**
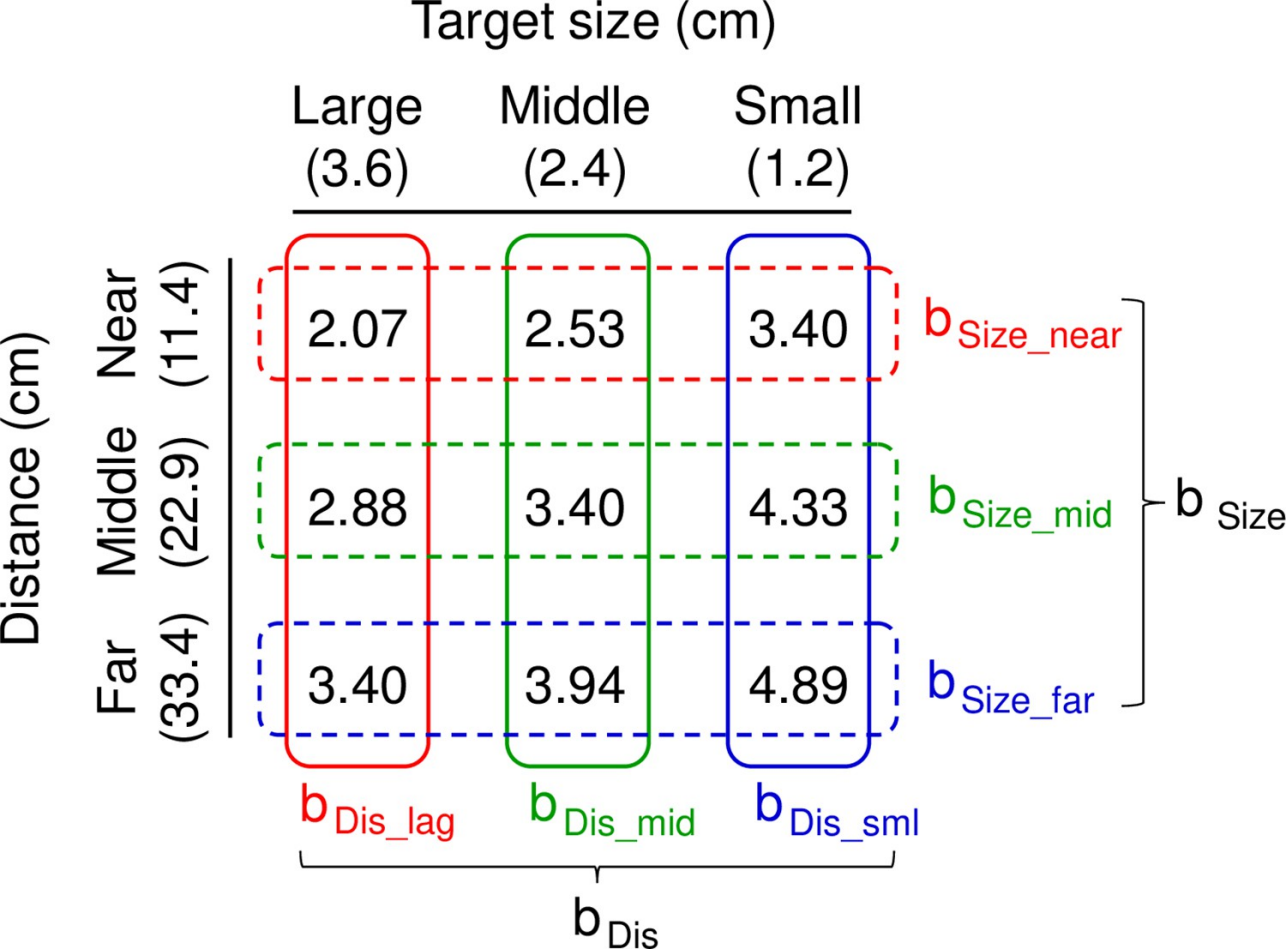
Index of difficulty calculated from each combined level of distance and target size. Combinations of the distance and target size produced a distinct value of the index of difficulty (ID). For each ID, the movement time (MT) of the pointing movement was obtained. At each level of the distance and target size, MTs were fitted by Mackenzie’s model, then the values of slope factor b in each level, indicating the effects of target size (b_Size_near_, b_Size_mid_, and b_Size_far_) and distance (b_Dis_lag_, b_Dis_mid_, and b_Dis_sml_) were obtained. Finally, b_Size_ and b_Dis_, showing generalized effect of target size and distance on MTs, was induced, respectively.

Participants engaged the upper limb-reaching movement toward the targets presented in the hemifield in front of the body. A white circle of the target was presented using custom-made software programmed with Visual Basic.NET running on a Windows PC. The targets with 3 different diameters (small: 1.2 cm, middle: 2.4 cm, and large: 3.6 cm) were presented at 15 locations in 5 directions (0°, 45°, 90°, 135°, and 180°) and 3 distances (near: 11.4 cm, middle: 22.9 cm, and far: 34.4 cm) from the home position (Fig 1A). Therefore, 45 conditions were selected based on target properties. Combinations of the target distance and target width produced a distinct value of the ID, as explained by Fitts’s law [1]. In this study, the ID defined by Mackenzie and Buxton [3] (Eq 2) was used.

$$ID=log_{2}(D/W+1)$$ (2)

where D indicates the distance between the starting point and center of the target and W indicates the size of the target. The nine IDs for all the D and W conditions are summarized in Fig 2.

Before the main trial, the participants practiced pointing movements 10 times toward randomly chosen targets. In the main trial (Fig 1B), one of the targets was blinked as pre-cueing, and the participant then placed the right index finger on the red circle of the home position and prepared for the initiation. A brief delay period, randomized between 1.5–3.5 s, was inserted to avoid the prediction of the movement’s initiation timing. After the delay, a target was presented on the display which indicated the initiation of the trial. The participant moved the index finger to the target under the instruction “point to the target as quickly as possible and touch inside but not necessarily on the center of the target.” When the index finger reached the target, the participant was asked to keep it on the target for a few seconds to clarify the termination of the trial. Forty-five trials with random order composed a block, and the participants engaged in 8 blocks (360 trials) in total. In some cases, the participants initiated the movement before the presentation of the target. In such cases, a beep sound was given and the trial was repeated.

### Data analysis

For the temporal analysis of pointing movements, MTs and RTs were evaluated. MT was defined as the time from the removal of the index finger to arrival at the target, whereas RT was defined as the time from the target presentation to the moment the index finger lost contact with the home position. The mean MTs and RTs for each condition were calculated for each participant. The data exceeding ±3 SD were excluded from the analysis [17].

The MTs were fitted by Mackenzie’s equation (Eq 1) for each direction and the slope factor b, indicating a change in the ratio of MTs in relation to changes in the distance and target size, was calculated. The values of slope factor b for large, middle, and small targets obtained by changing the distance (b_Dis_lag_, b_Dis_mid_, and b_Dis_sml_) were averaged, and then b_Dis_ showing generalized effect of distance on MTs was induced (Fig 2). Similarly, the values of slope factor b for near, middle, and far targets obtained by changing the target size (b_Size_near_, b_Size_mid_, and b_Size_far_) were averaged, and then b_Size_ showing generalized effect of target size on MTs was induced.

For statistical analysis, a three-way analysis of variance (ANOVA) was performed for the values of MTs and RTs with the factors of direction (5), distance (3), and target size (3). Similarly, a two-way ANOVA was performed for the slope factor b with the factors of direction (5) and manipulating parameter (2: distance or target size, which correspond to b_Dis_ and b_Size_, respectively). For post-hoc comparisons, Bonferroni’s test was used. The significance level was set at 0.05.

## Result

Successful data were obtained from 4293 trials (99.4%) under all conditions. Other data from 27 trials (0.4%) were eliminated from the analysis because of technical problems such as marker hiding and inactivation of the sensor.

### Movement time

**Fig 3:**
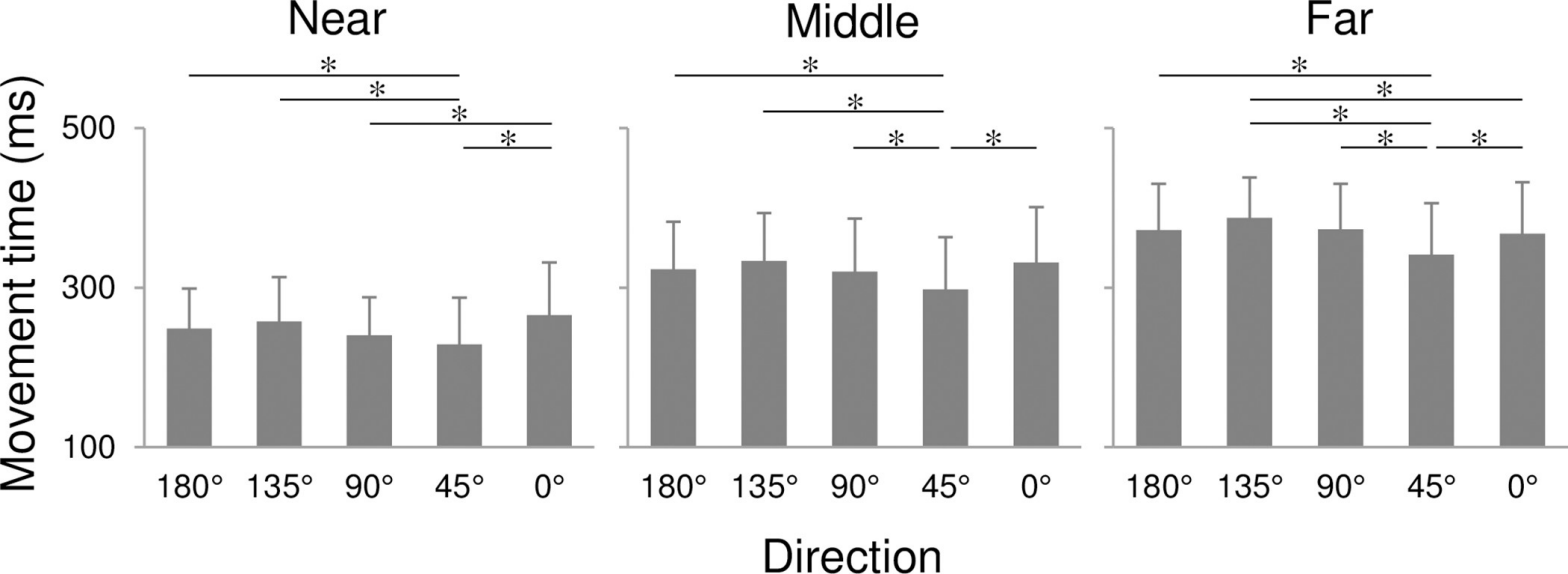
Movement time relative to movement direction. Error bars show standard error. *: p < 0.05.

A three-way ANOVA for MTs revealed main effects in direction (F(4, 44) = 20.97, p < 0.01, η^2^ = 0.026), distance (F(2, 22) = 461.83, p < 0.01, η^2^ = 0.394) and target size (F(2, 22) = 13.15, p < 0.01, η^2^ = 0.048). In addition, a first-order interaction was observed in the factors of direction and distance (F(8, 88) = 4.47, p < 0.01, η^2^ = 0.004). Multiple comparisons of the main effect of target size revealed that the MTs for a target size of 1.2 cm were significantly greater than those for 2.4 cm and 3.6 cm (both p < 0.05). Meanwhile, a multiple comparison for the first-order interaction observed between distance and direction indicated that the MTs differed significantly according to the direction at each distance, with MTs at 45° being the smallest (Fig 3).

### Slope factor b

**Fig 4:**
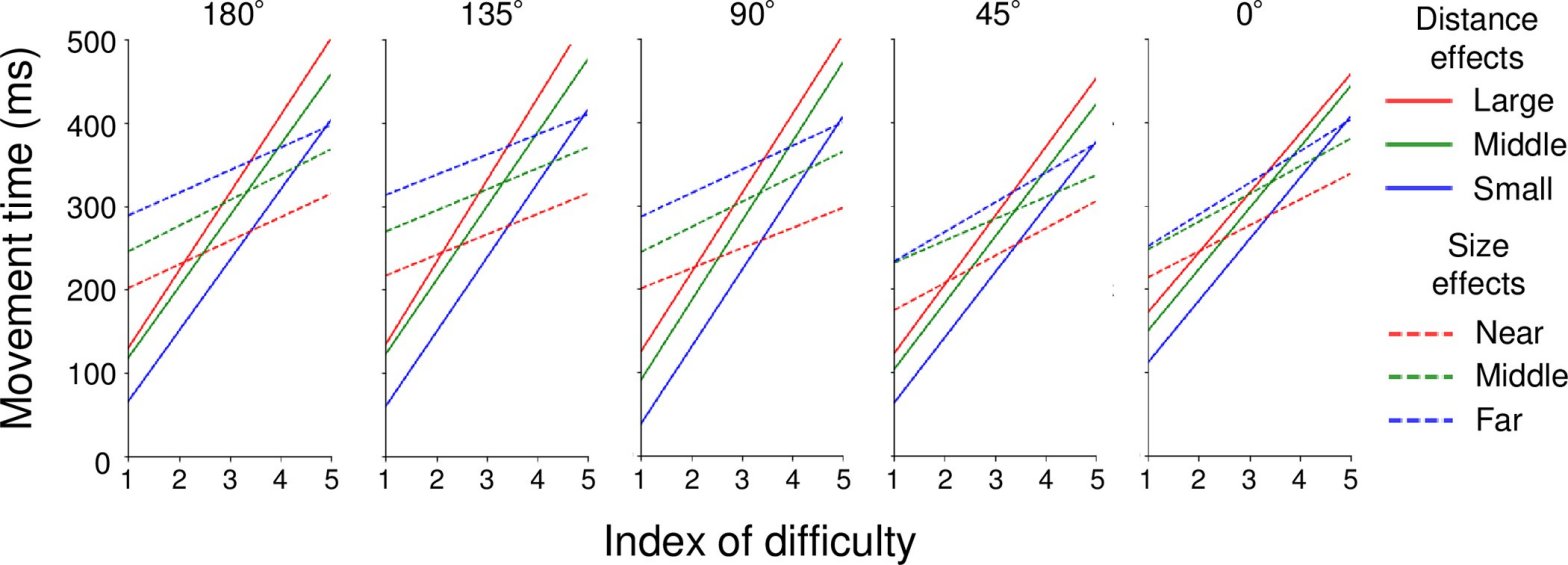
Movement time in each movement direction relative to index of difficulty. The movement time according to the change in distance is shown by solid lines, whereas that according to the target size is shown by broken lines. The color and type of each line correspond to those shown in Fig 2.

**Fig 5:**
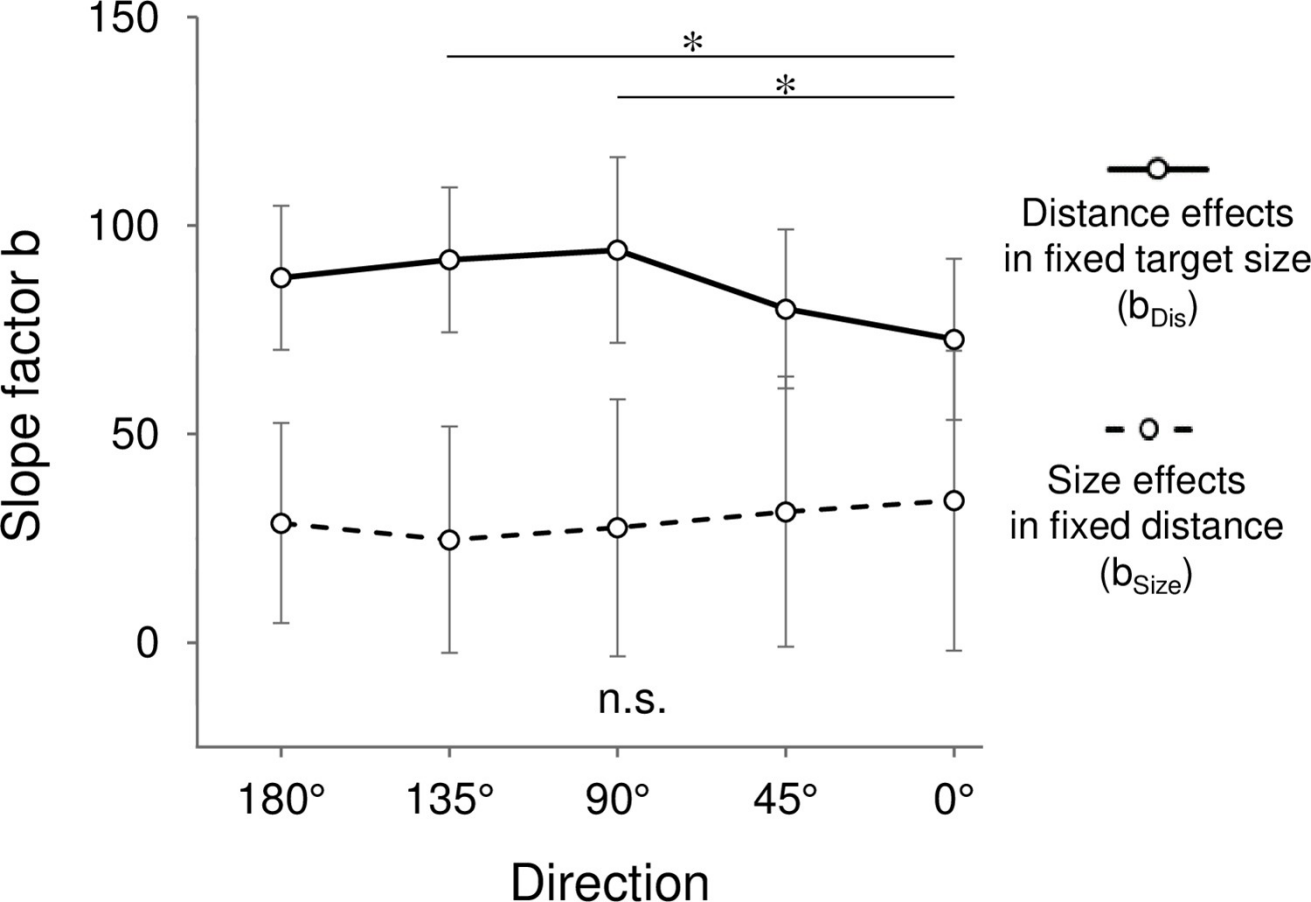
Slope factor b relative to movement direction. Error bars show standard error. *: p < 0.05.

Fig 4 shows the fitted lines of MTs relative to ID with the change in distance (solid lines) or target size (broken lines) for each condition. The slope of each line indicates the slope factor b for each condition across participants. A two-way ANOVA for the slope factor b revealed a main effect of the manipulating parameter (F(1,11) = 100.34, p < 0.01, η^2^ = 0.594). A post-hoc test revealed that the slope factor b due to the change in distance (b_Dis_) was significantly larger than that of the target size (b_Size_). In other words, the modification of the distance was more effective for MTs than that of the target size. Meanwhile, an interaction between the direction and manipulating parameter (F(4,44) = 7.65, p < 0.01, η^2^ = 0.023) was observed. A multiple comparison between the directions for the level of b_Dis_ indicated that the b_Dis_ for 0° was significantly smaller than that for 90° and 135° (p < 0.05, Fig 5). These results indicated that the change in MT relative to the change in distance depended on the direction, and 0° showed the smallest change.

### Reaction time

**Fig 6:**
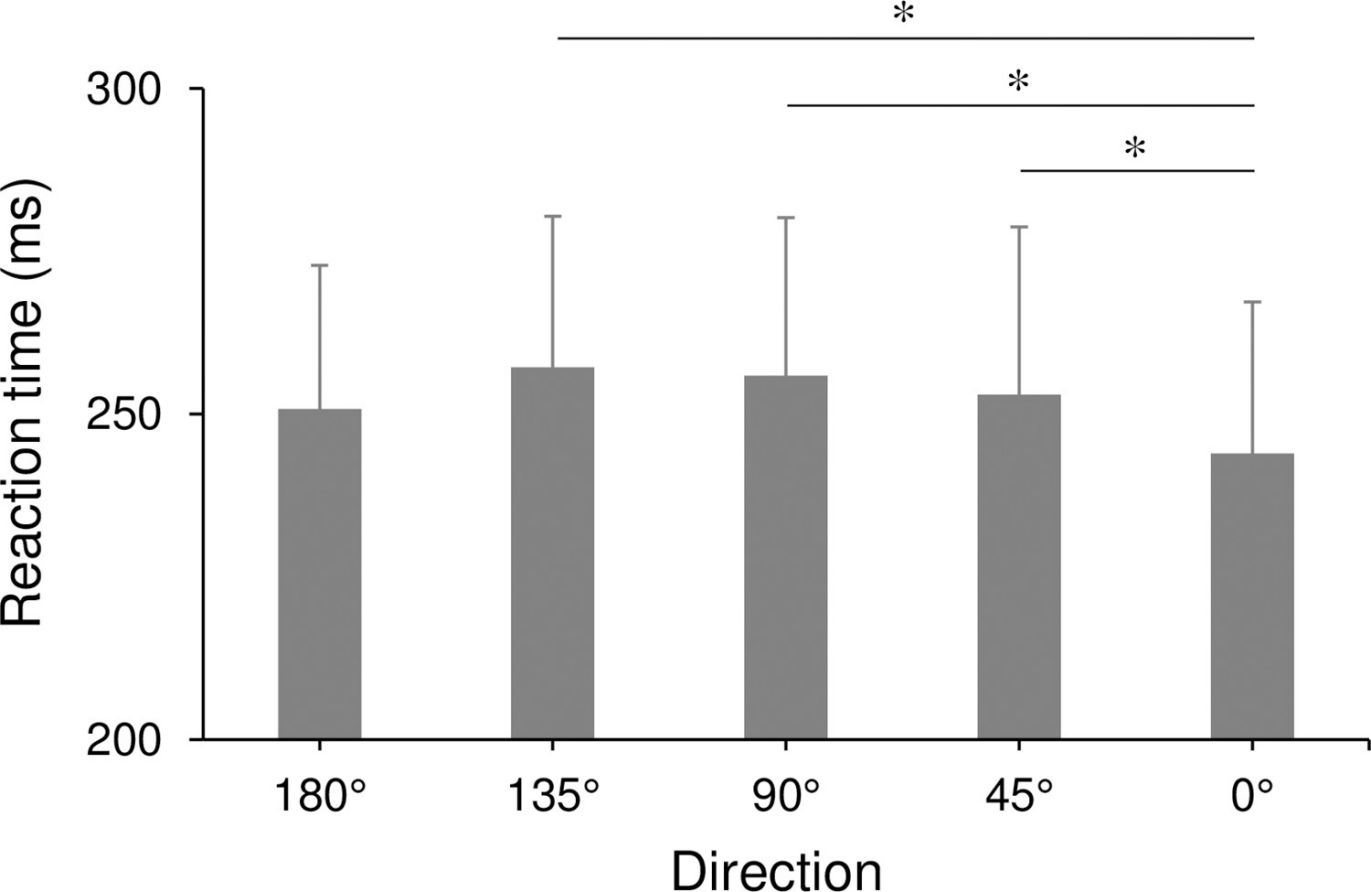
Reaction time relative to movement direction. Error bars show standard error. *: p < 0.05.

A three-way ANOVA of RTs revealed main effects of direction (F(4,44) = 8.41, p < 0.01, η^2^ = 0.037), distance (F(2,22) = 43.24, p < 0.01, η^2^ = 0.064), and target size (F(2,22) = 40.51, p < 0.01, η^2^ = 0.043). No interaction effects were observed among those factors. A multiple comparison of directions showed that the RTs at 0° was significantly smaller than that at 45°, 90°, and 135° (all p < 0.05; Fig 6). For the factor of target size, a multiple comparison indicated that RTs in the target size of 1.2 cm was significantly larger compared to that of 2.4 cm and 3.6 cm, and that of 2.4 cm was significantly larger compared to that of 3.6 cm. For the factor of distance, a multiple comparison revealed that RTs in 34.4 cm was significantly larger compared to that of 22.9 cm and 11.4 cm, in addition, that of 22.9 cm was significantly larger compared to that of 11.4 cm.

## Discussion

This study aimed to elucidate the role of the direction in modulating the performance of upper limb movements that directly manipulated objects, and the effect of the interactions between direction and distance and between direction and target size on the performance. We examined MTs for arm-pointing movements and induced the slope factor b, which showed the ratio of change for MTs in response to changes in the distance and target size for each direction. We found that MTs significantly varied according to the direction for right-forward being the smallest. Meanwhile, the slope factor b for distance was larger than that for target size, and that for distance was smaller in the rightward direction than that in the forward or left-forward directions.

### Anisotropy of arm-pointing movements

In this study, MT at 45° (right-forward direction) was smaller than MT at 135° (left-forward direction) and 180° (leftward direction). Such an anisotropy in MT with predominance ipsilateral side had been observed in previous studies with upper limb movements [18] or a mouse [8]. In contrast, other studies on pointing movements with a mouse showed that the right-forward direction had relatively longer MTs than any other direction [3,19–21]. Such a discrepancy in MTs depending on the direction could be partly attributed to the shapes of the targets. Gordon et al. [8] used circular targets, whereas others used square targets, which might differ in the task demand for adjusting the cursor to the target at the last stage of the movement. However, the emergence of anisotropy in MTs can also be related to mechanical factors of movement. Takeda et al. [16] examined effects of kinetic properties during reaching movements and showed that the time required for movement was significantly influenced by the motor cost such as moment of inertia, joint torque, and signal-intensity-dependent noise. Additionally, Guigon et al. [12] showed that signal-dependent noise was influenced by the movement direction. Therefore, it is suggested that muscles and joints with different kinetic properties are driven in each direction of movement, which in turn affects performance depending on the direction.

Some studies attempted to combine the ability of speed and accuracy into a single index and showed that its ability depended on the individual or the task [22,23]. Such an evaluation could also be applied to arm-pointing movements in various directions. The combined ability of speed and accuracy could differ according to the direction, with its higher score in the right-forward direction compared to the scores in other directions. Since the present study focused more on the temporal property, i.e., the component of speed, of arm-pointing movements, future studies exploring its spatial property, i.e., the component of accuracy, would elucidate more details about the direction-dependent motor ability of the upper limb.

According to the results, RTs changed depending on the direction (Fig 6). In that, the motor cost for the initiation of the upper limb movement might be involved since it depended on the movement direction [16], which was also suggested for MTs. In addition, the anisotropy observed in RTs could also be due to a cognitive factor that depended on the movement direction. Ishihara et al. [15] examined the RTs of pointing tasks toward different target locations and elucidated that the time required for motor planning depended on the target location with a shorter time toward the rightward direction and a relatively longer time toward the forward and left-forward directions. Such an anisotropy could also be involved in the RTs observed in this study.

From another viewpoint, the central nervous system (CNS) is known to represent near space around the body, i.e. peripersonal space (PPS), in relation to one’s ability of action. The PPS for right-handers showed anisotropic features, with the dominant hand side being smaller and the non-dominant hand side being larger [24]. The authors suggested that the anisotropy observed was reflecting the necessity of protection against a harmful object more quickly on the non-dominant hand side. Similarly, the larger MTs and RTs in the left-forward direction for right-handers observed in this study might be related to a larger PPS to avoid danger on the non-dominant hand side of the hemifield. As a result, it is suggested that the anisotropy of motor performance observed in this study is attributable to that of spatiotemporal properties in both motor and cognitive functions.

### Effects of distance and target size on arm-pointing movements depending on the direction

While the amount of MT suggests the difficulty of movement, that of slope factor b suggests its ratio of change relative to a certain parameter. We observed that the slope factor b for the distance was significantly larger than that for the target size (Fig 5). In other words, changes in ID caused by manipulating the distance were more effective for MTs than those caused by manipulating the target size. This result is consistent with previous studies that examined MTs in pointing movements with a finger [13,14]. It is known that pointing movement with a finger decreases its spatial accuracy and shorten MTs compared to that using input devices, such as a mouse or pen, especially for small targets [25]. This phenomenon is considered to be caused by the situation that the finger hides visual information of the target immediately before arrival [26]. Therefore, the wide surface of the finger arrives roughly around the target, and the spatial accuracy required for the pointing movement is reduced. Consequently, pointing movements using a finger without input devices lower the cognitive cost for operating the movements [27]. These factors are thought to affect the speed-accuracy trade-off, resulting in shorter MTs for small targets. In this study, the MTs for the distance effects in small targets (blue solid lines in Fig 4) were shorter than those in the middle (green solid line) and large (red solid line) targets. As the effect of distance was stronger than that of the target size, it seemed that the MTs for the small target with near distance were shorter than those for the large (middle) target with far (middle) distance at a certain ID.

The result of this study showed that the slope factor b of distance effects (b_Dis_) was the smallest in the rightward (0°) direction (Fig 5) and was significantly smaller compared to those for the forward (90°) and left-forward (135°) directions. The smallest change of MTs depending on the distance in the rightward direction was directly confirmed in Fig 3 with MTs being the largest at near distance while the second smallest at far distance among all directions. Such direction-specific changes of MTs could be related to two possible reasons. First, pointing movements toward the rightward direction required little trunk movements, compared to those for other directions. Bertucco et al. [28] examined MTs during a pointing task in standing participants in relation to the body movement. They set two targets with identical ID, near-small and far-large targets, and observed that the MTs for far-large targets were larger than those for near-small targets. They suggested that the movements in both conditions contributed differently to the movements of the upper limb and trunk. For far-large targets, trunk movements with larger inertia were recruited more during the task than for near-small targets. Similarly, in this study, pointing movements in the rightward direction (0°) involved more arm movements at any distances, whereas the forward (90°) and left-forward (135°) directions involved more trunk movements, although this was not quantitatively assessed. It is suggested that such a different involvement of body segments might have induced a smaller change of MTs in the rightward direction compared to those in the forward and left-forward directions, which resulted in the anisotropy observed in b_Dis_.

Second, the involvement of joints for pointing movements toward the rightward direction was considered to significantly vary according to the distance. For the near target in the rightward direction, the pointing movement required extending the shoulder joint, which was not the case for other directions. Such a movement for the right-near target might have required more motor costs and induced longer MTs. Meanwhile, for the far target in the rightward direction, the pointing movement required not only extension but also external rotation of the shoulder joint. Such a movement got closer to that in the right-forward (45°) direction, in which the smallest MTs among all directions were observed, and might have contributed to produce the second shortest MTs. As a result, the pointing movements in the rightward direction was considered to require the unique joint involvement depending on the distance, which might cause the smaller value of slope factor b for distance, compared to other directions.

On the other hand, the slope factor b for target size was not clearly observed in relation to the direction. This could be because pointing movements with a finger decreased spatial accuracy and shortened MTs. If input devices were used for pointing movements, the slope factor b for target size might be depended more on the movement direction.

To date, a limited number of studies have investigated the properties of Fitts’s law in relation to the movement direction. Murata and Iwase [11] examined MTs in relation to the movement direction and extended Fitts’s law by adding a parameter concerning it. They observed that MTs for arm-pointing movements to the targets on a vertical plane showed anisotropy, and introduced a modified version of Mackenzie’s model. Although this study examined MTs in relation to the movement direction as like Murata and Iwase [11] did, there were differences in several aspects. Murata and Iwase [11] placed targets on a vertical plane while this study placed them on a horizontal plane. In addition, our study distinguished the effects of distance and target size on MTs, whereas they did not. Despite such differences, the slope factor b for the distance effects indicated a modest curve (Fig 5), which showed the dependence of the movement direction on Fitts’s law, and was similar to the anisotropy of MTs observed in Murata and Iwase [11].

These effects of the distance and target size on upper-limb movements in various directions around the body are needed to be modeled more precisely. Further study may be required to elucidate how Fitts’s law should be extended by adding parameters concerning the movement direction.

## Conclusion

This study examined the variations in temporal properties of the speed-accuracy trade-off for upper limb movements according to the movement direction, distance, and target size. The results showed that the MTs exhibited anisotropy in the hemifield with the right-forward direction being the smallest. In addition, the slope factor b, due to the change in distance, was smaller in the rightward direction than those in the forward and left-forward directions. These results suggest that the degree of difficulty of upper limb movements expands heterogeneously in the workspace around the body. Furthermore, the effects of parameters composing Fitts’s law vary largely in the horizontal plane depending on the movement direction.

## Supporting information

S1 Table Coefficient of determination (R^2^) of each effect for each participant. (PDF)
