# Supplementary material for: Temporal properties of the speed-accuracy trade-off for arm-pointing movements in various directions around the body
Source: PLoS One. 2023 Sep 21;18(9):e0291715. doi: 10.1371/journal.pone.0291715 (PMC10513193; doi:10.1371/journal.pone.0291715)
Supplement: S1 Table — (PDF) [file pone.0291715.s001.pdf]

S1A. Table. Coefficient of determination (R^2) in distance effects for each participant

	180°			135°			90°			45°			0°		
	Large	Middle	Small	Large	Middle	Small	Large	Middle	Small	Large	Middle	Small	Large	Middle	Small
Max	0.900	0.833	0.814	0.876	0.849	0.853	0.895	0.867	0.835	0.850	0.780	0.761	0.818	0.786	0.775
Median	0.680	0.627	0.580	0.720	0.700	0.615	0.760	0.678	0.632	0.566	0.563	0.487	0.478	0.564	0.571
Min	0.469	0.462	0.378	0.439	0.189	0.356	0.363	0.432	0.414	0.359	0.439	0.281	0.090	0.271	0.183

S1B. Table. Coefficient of determination (R^2) in size effects for each participant

	180°			135°			90°			45°			0°		
	Near	Middle	Far	Near	Middle	Far	Near	Middle	Far	Near	Middle	Far	Near	Middle	Far
Max	0.439	0.452	0.534	0.648	0.529	0.694	0.546	0.614	0.873	0.425	0.747	0.747	0.704	0.781	0.687
Median	0.120	0.095	0.096	0.052	0.080	0.088	0.082	0.161	0.086	0.144	0.059	0.181	0.095	0.096	0.107
Min	0.018	0.000	0.001	0.002	0.001	0.003	0.002	0.000	0.006	0.000	0.001	0.002	0.007	0.002	0.009
